# More Evidence for Isolation of the Left Atrial Free Wall During the Convergent Procedure in Atrial Fibrillation

**DOI:** 10.19102/icrm.2018.091006

**Published:** 2018-10-15

**Authors:** Andy C. Kiser

**Affiliations:** ^1^East Carolina Heart Institute at East Carolina University, Greenville, NC, USA; ^2^Department of Cardiovascular Sciences, Brody School of Medicine, Greenville, NC, USA

**Keywords:** Atrial fibrillation, convergent, hybrid, surgery

The report by Nagarakanti et al.^1^ in this issue of *The Journal of Innovations in Cardiac Rhythm Management* examines the role of posterior left atrial isolation during hybrid convergent procedure in seven patients. In a similar vein, we previously examined the impact of posterior left atrial wall isolation on the restoration of sinus rhythm.^2^ Our examination of 57 patients with persistent or high-burden paroxysmal atrial fibrillation (27 convergent; 30 endocardial catheter-only) reconfirmed the difficulty in obtaining acute electrical isolation of the posterior LA (51.9% in convergent patients; 23.3% in catheter-only patients) and failed to prove a statistical benefit regarding the treatment of recurrent arrhythmias. We have, however, seen the favorable resolution of atrial fibrillation in our convergent patients who demonstrated elimination of posterior left atrial signal during the procedure.^3^

The embryological and fibrotic considerations Nagarakanti et al.^1^ present and the procedural success of the Cox maze procedure lend convincing support for the isolation of the left atrial free wall in patients with advanced atrial fibrillation. The creation of contiguous, transmural ablation lines that electrically isolate the left atrium and pulmonary veins acutely and chronically is likely to improve procedural outcomes. The Epi/Endo Ablation for Treatment of Persistent Atrial Fibrillation (CONVERGE) trial (NCT01984346), which aims to study the treatment of symptomatic persistent AF patients who are refractory or intolerant to at least one class I or class III antiarrhythmic drug via the use of the EPi-Sense^®^-AF Guided Coagulation System with VisiTrax^®^ (AtriCure, Mason, OH, USA), has almost completed enrollment and is expected to provide additional insight regarding this question.
